# Selection of a reference gene for studies on lipid‐related aquatic adaptations of toothed whales (*Grampus griseus*)

**DOI:** 10.1002/ece3.8354

**Published:** 2021-11-26

**Authors:** Jayan D. M. Senevirathna, Ryo Yonezawa, Taiki Saka, Yoji Igarashi, Noriko Funasaka, Kazutoshi Yoshitake, Shigeharu Kinoshita, Shuichi Asakawa

**Affiliations:** ^1^ Laboratory of Aquatic Molecular Biology and Biotechnology Department of Aquatic Bioscience Graduate School of Agricultural and Life Sciences The University of Tokyo Tokyo Japan; ^2^ Department of Animal Science Faculty of Animal Science and Export Agriculture Uva Wellassa University Badulla Sri Lanka; ^3^ Department of Life Sciences and Chemistry Graduate School of Bioresources Mie University Mie Japan; ^4^ Department of Life Sciences Graduate School of Bioresources Mie University Mie Japan

**Keywords:** cetacean, gene expression, gene stability, qPCR, reference gene, transcriptomics

## Abstract

Toothed whales are one group of marine mammals that has developed special adaptations, such as echolocation for predation, to successfully live in a dynamic aquatic environment. Their fat metabolism may differ from that of other mammals because toothed whales have acoustic fats. Gene expression in the metabolic pathways of animals can change with respect to their evolution and environment. A real‐time quantitative polymerase chain reaction (RT‐qPCR) is a reliable technique for studying the relative expressions of genes. However, since the accuracy of RT‐qPCR data is totally dependent on the reference gene, the selection of the reference gene is an essential step. In this study, 10 candidate reference genes (*ZC3H10*, *FTL*, *LGALS1*, *RPL27*, *GAPDH*, *FTH1*, *DCN*, *TCTP*, *NDUS5*, and *UBIM*) were initially tested for amplification efficiency using RT‐qPCR. After excluding *DCN*, the remaining nine genes, which are nearly 100% efficient, were selected for the gene stability analysis. Stable reference genes across eight different fat tissue, liver, and muscle samples from *Grampus griseus* were identified by four algorithms, which were provided in Genorm, NormFinder, BestKeeper, and Delta CT. Finally, a RefFinder comprehensive ranking was performed based on the stability values, and the nine genes were ranked as follows: *LGALS1* > *FTL* > *GAPDH* > *ZC3H10* > *FTH1* > *NDUS5* > *TCTP* > *RPL27* > *UBIM*. The *LGALS1* and *FTL* genes were identified as the most stable novel reference genes. The third‐ranked gene, *GAPDH*, is a well‐known housekeeping gene for mammals. Ultimately, we suggest the use of *LGALS1* as a reliable novel reference gene for genomics studies on the lipid‐related aquatic adaptations of toothed whales.

## Graphical abstract

The lipid metabolism of toothed whales is an interesting topic to study. RT‐qPCR‐based gene expression and stability study was conducted using various lipid tissues in Risso's dolphin and the *LGALS1* gene was identified as the most stable reference gene for further transcriptomic studies.

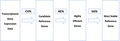

## 1 INTRODUCTION

The *Grampus griseus* (Risso's dolphin) is a toothed whale species in the order Cetacea that exhibits cosmopolitanism (Gaspari et al., 2007; Gaspari & Natoli, 2012). *Grampus griseu* is the only species in the genus *Grampus*, the fifth‐largest member of the family Delphinidae (Baird, 2009), and part of the subfamily Globicephalinae. Species in this subfamily could be cryptic species by evolutionary imperative (Thompson et al., 2013). Even within the subfamily Globicephalinae, *G*. *griseus* display a 0.022 distance from other species in the phylogeny of complete mitochondrial genomes (Senevirathna et al., 2021). Evolutionary forces and environmental factors may affect the shaping of rare dolphin populations. These major evolutionary forces consist of genetic mutations, natural selection, genetic drift, and gene flow (Saeb & Al‐Naqeb, 2016). Previous research has examined the evolution of fitness rates due to the interaction of social and genetic factors in a bottlenose dolphin population (Frère et al., 2010). In addition, an analysis of dolphin genomes has observed an adaptive evolution of nervous system genes and a slow metabolic rate (McGowen et al., 2012). Therefore, evolutionary studies on metabolic genes are vital to identify special aquatic adaptations of these cetaceans.

We theorize that genes involved in lipid metabolism may have a stronger evolutionary influence on cetaceans. Furthermore, evolutionary or selective pressure can affect gene evolution for cetacean aquatic adaptations. The intake of resources from the living environment impacts the adaptive evolution of metabolism and diversification and the synthesis of fatty acids in marine animals (Twining et al., 2021). One important ecological feature of the Risso's dolphins is their carnivorous feeding behavior (Baird, 2009). Feeding mainly on squids, such as cephalopods, is a common habit of toothed whales, which may relate to their lipid metabolism.

Generally, toothed whales exhibit unique metabolic adaptations, such as heat regulation to survive in the cold marine environment (Yuan et al., 2021), and metabolic changes can be caused by anthropogenic and climatic factors. Toothed whales consist of several types of fat deposits, including the acoustic fats in the head region, which are involved in the special aquatic adaptation of echolocation. These acoustic fats predominantly contain unusual wax esters and triglycerides (Koopman et al., 2006; Norris, 1974). However, their fatty acid composition varies based on the species, age, and type of tissue (Koopman, 2018). There is an increasing interest in studying toothed whales at the genomic and transcriptomic levels to reveal potential genes and distinctive metabolic pathways that are implicated in this specialized process of fat‐metabolism‐related aquatic adaptations. A recent supportive study has identified positively selected genes for lipid metabolism in Cetacea as well as unique features for examining functional modifications of multiple genes (Endo et al., 2018). However, environmental stressors can alter lipid metabolism pathways and directly affect lipid oxidation in animals (Koelmel et al., 2020). Lipidomics is also relevant in environmental toxicology research; it can be applied to lower organisms and higher organisms, such as mammals, and is an ideal molecule for analyzing animal health (Aristizabal‐Henao et al., 2020). Another study has confirmed the importance of identifying a stable reference gene to clarify biomarkers for exploring environmental stresses on the cellular adaptations of animals using *Nacella* spp. under different environmental conditions (Koenigstein et al., 2013). Therefore, environmental lipidomics represents the next level of molecular application in wild animal research, and investigations of lipid‐related reference genes in toothed whales will be key for various future ecological studies concerning toxicology and lipidomics.

A reverse transcription quantitative real‐time polymerase chain reaction (RT‐qPCR) has numerous biological applications for targeted gene expression and is especially advantageous for non‐model species (Fassbinder‐Orth, 2014). This technique requires an accurate and reliable reference or housekeeping gene (HKG) to normalize specific gene expression data for relative quantification (Almeida‐Oliveira et al., 2017). In certain tissues of a particular species, the expression level of genes may differ significantly compared with a selected reference gene (Radonić et al., 2004). Therefore, it is crucial to identify a reliable reference gene before conducting a comparison with the genes of interest. Specifically, in toothed whales, the adipose tissue metabolic pathway is still not clear, and supportive lipid metabolism genes have not been investigated at the transcriptomic level. It is possible that there are unique de novo biosynthesis pathways of lipids in toothed whales for various ecological adaptations (Koopman, 2018). Therefore, identifying a stable reference gene is necessary for future RT‐qPCR assays of the functional genomics of lipid metabolic pathways to understand molecular evolutionary implications.

Frequently used reference genes for mammals include 18S ribosomal RNA (*18S rRNA*), glyceraldehyde‐3‐phosphate dehydrogenase (*GAPDH*), 28S ribosomal RNA (*28S rRNA*), β actin (*ACTB*), and succinate dehydrogenase complex subunit A (*SDHA*). These genes present a high level of expression in various mammalian tissues. In skin biopsies and blood samples from several species of marine mammals, the following genes have been evaluated as HKGs: phosphoglycerate kinase 1 (*PGK1*), hypoxanthine phosphoribosyl transferase 1 (*HPRT1*), and ribosomal protein L4 (*RPL4*) (Chen et al., 2015); ribosomal protein L8 (*RPL8*) (Buckman et al., 2011); *GAPDH* (Mancia et al., 2008; Spinsanti et al., 2006); tyrosin 3‐monooxygenase/tryptophan 5‐monooxygenase activation protein zeta (*YWHAZ*) (Beineke et al., 2004, 2007; Muller & Woods, 2013); ribosomal protein S9 (*RPS9*) (Sitt et al., 2008, 2010; Viscarra & Ortiz, 2014); and ribosomal protein S18 (*RPS18*) (Martinez‐Levasseur et al., 2013). However, there is a lack of selection of reference genes for fat tissues in toothed whales.

**FIGURE 1:**
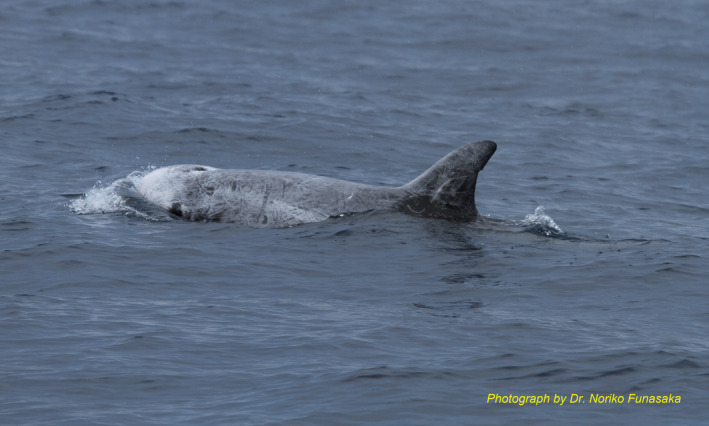
Study species, Risso's dolphin (*Grampus griseus*)

**FIGURE 2:**
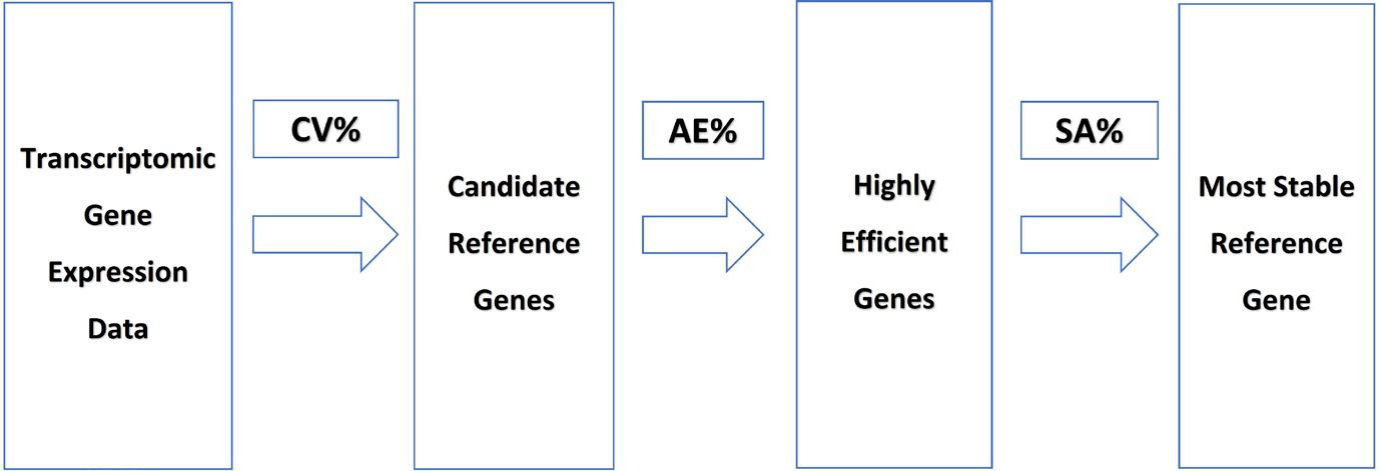
Diagram of experimental design. CV%, coefficient of variance calculated by FPKM values; AE%, amplification efficiency based on RT‐qPCR; SA%, stability analysis by statistical algorithms

The present study used tissue samples from Risso's dolphins (Figure 1). The experimental design emphasized the identification of a novel reference gene for adipose tissues (Figure 2). Ten candidate genes were initially selected based on transcriptomic fragments per kilobase of transcript per million mapped reads (FPKM) values, the coefficient of variance (CV), gene function, and relevance to metabolism. Then, the identified genes were evaluated for amplification efficiency through RT‐qPCR. Only the most efficient genes were retained for further stability analysis using eight types of fat tissues, liver tissue, and muscle tissue with the aim of identifying the most stable reference gene. The null hypothesis, which predicted no significant difference in selected reference gene stability, was tested by four statistical algorithms. This work ultimately validates stable novel genes in the selected tissue types. Future research can utilize these findings for gene expression analysis in marine mammal species and particularly to study fat metabolism in toothed whales.

## 2 MATERIALS AND METHODS

### 2.1 Sample collection

Ten types of tissue from three male Risso's dolphins (sample ID: 19TK409, 19TK410, and 19TK411) were received from the Taiji Fisheries Association with the cooperation of the biological surveys of the National Research Institute of Far Seas Fisheries and the Japan Fisheries Research and Education Agency. The tissues included four parts of the melon, two types of jaw fat, two parts of blubber, liver, and muscle. All of these tissue types are non‐reproductive; therefore, the use of only male samples for this analysis does not risk any sex‐related bias. These tissues were preserved in an RNAlater solution at the site before being transported to the laboratory, where they were stored at −80°C until RNA extraction.

### 2.2 RNA extraction, sequencing, and cDNA synthesis

The RNA was extracted by the RNAiso Plus (total RNA extraction reagent) method according to the manufacturer's instructions (Code 9108, Takara Bio Inc., Japan). In summary, 20 mg of each sample was homogenized for 2 min at 2616 *g* by a tissue homogenizer (Precellys^®^ 24) with RNAiso plus and beads. The homogenized samples were transferred to a centrifuge tube and incubated for 5 min at room temperature (RT). Then, the samples were centrifuged at 12,000 *g* for 5 min at 4°C, and supernatants were collected in new tubes. Chloroform was added to each sample in the amount of one‐fifth of the amount of RNAiso plus, and the solution was mixed until it became milky. The tubes were kept at RT for 5 min and subsequently centrifuged at 12,000 *g* for 15 min at 4°C. The top layer, which contained RNA, was transferred to new tubes. The RNA pellets were precipitated by adding one‐half of the amount of isopropanol and centrifuging at 12,000 *g* for 10 min at 4°C. The precipitates were washed with 75% cold ethanol and centrifuged at 7500 *g* for 5 min at 4°C. The RNA pellets were dried for several minutes and dissolved with 100 µl of RNase‐free water.

The cDNA library sequences were generated through the low sample protocol of the Trueseq Stranded mRNA sample preparation guide for Illumina^®^ Hiseq (Illumina, San Diego, CA, USA) following the manufacturer's recommendations. The libraries were used for quality control analysis and quantification by the Agilent Tape Station 2200 system (Agilent Technologies, Santa Clara, CA, USA). Finally, the libraries were normalized and pooled into a single library. This library was loaded into a flow cell for clustering with surface‐bound oligos complementary to the library adapters, and bridge amplification was carried out to prepare clonal clusters for sequencing by the Illumina HiSeq platform (Illumina, San Diego, CA, USA). Paired‐end raw reads were generated by RTA2 and bcl2fastq2‐v2‐20.0 (Macrogen NGS service).

The RNA clean‐up was conducted with the NucleoSpin RNA Clean‐up XS kit (MACHEREY‐NAGEL, Düren, Germany) following the protocol (View, 2014). First, DNA in the crude RNA extracts was digested by adding a one‐tenth mixture of rDNase (Macherey‐Nagel, 740963) and a reaction buffer and incubating for 10 min at 37°C. An equal volume of buffer RCU was then added to each sample and mixed for 2 × 5 s. The solutions were added to NucleoSpin RNA XS columns with collection tubes and centrifuged for 30 s at 11,000 *g*. Next, 400 µl of buffer RA3 was added to each tube and centrifuged for 30 s at 11,000 *g*. The flow‐through was discarded, and 200 µl of buffer RA3 was introduced to the columns and centrifuged for 2 min at 11,000 *g* to dry the membrane containing purified RNA. Subsequently, each column was placed in a nuclease‐free collection tube (1.5 ml, supplied), and RNA was eluted in 30 µl of RNase‐free water by centrifuging at 11,000 *g* for 30 s. The RNA concentrations were checked with the Qubit^®^ RNA Assay Kit in Qubit^®^2.0 Fluorometer (Life Technologies, Carlsbad, CA, USA), and the RNA quality was verified by means of the Agilent 2200 Tape Station (Agilent Technologies, Santa Clara, CA, USA). The RNA samples were stored at −80°C for further use.

First‐strand cDNA was synthesized for each RNA sample using the PrimerScript™ RT master mix kit (Perfect Real Time) (Code RR037A, Takara Bio Inc., Japan) with 2 µl of master mix, up to 500 ng of purified RNA, and RNase‐free water in a 10 μl solution on ice followed by incubation on a thermal cycler at 37°C for 15 min and 85°C for 5 s. The Qubit was employed to check the cDNA concentrations for all samples, and the presence of cDNA was checked by normal PCR using a selected primer before RT‐qPCR. The cDNA samples were stored at −20°C until further use.

### 2.3 Selection of candidate reference genes using RNA‐seq data and designation of primers

Raw RNA sequencing data were obtained from the company Macrogen Japan Corp. (order no. 1908JNHX‐0031). To obtain high‐quality clean reads, raw reads were used to trim adapters and filtered by removing the poly‐A sequence that contained more than five bases at the 3′ end and had a Q Phred quality score of 20 or less. Then, clean reads were mapped to the reference common bottlenose dolphin (*Tursiops truncates*) genome sequence (GCF_001922835.1_NIST_Tur_tru_v1, downloaded from NCBI; Martinez‐Viaud et al., 2019), filtered by PRINSEQ 0.20.4 (Schmieder & Edwards, 2011), aligned with HISAT2 2.1.0 (Kim et al., 2019), and annotated to the gene transfer format of the same genome by StringTie 1.3.4 (Pertea et al., 2015) in the RNA‐seq analysis PortablePipeline‐win‐v0.9c 2019 (https://github.com/c2997108/OpenPortablePipeline). Cufflink was used for mapped read splicing, and reads were then compared with genomic annotation information to explore new genes (Trapnell et al., 2010). Furthermore, the number of reads mapped to a particular gene was calculated with a ballgown algorithm (Frazee et al., 2015) in the pipeline. Finally, the gene count data and normalized FPKM values were obtained.

Ten candidate reference genes were selected, including novel genes, that displayed a low CV value, gene function in metabolism, and high FPKM values in all tissues. These candidate genes were chosen based on unpublished RNAseq data, which are available from the corresponding author upon request. Table 1 specifies the gene IDs, gene names, and functions of the candidate reference genes. The expression stability of the selected genes was calculated and summarized as follows (Wang et al., 2014). First, using the FPKM values of gene expression in 30 samples, mean expression values and standard deviations (SDs) were calculated for each gene. Second, the CV of each gene was ranked according to the CV value (Table 2).

**TABLE 1:** Information about selected candidate reference genes

Gene	Name	Functions by GeneCards
*ZC3H10*	Zinc finger CCCH‐type containing 10	Specifically regulates miRNA biogenesis
*FRIL/FTL*	Ferritin light chain	Stores iron in a soluble and nontoxic state
*LEG1/LGALS1*	Galectin 1	Modulates cell–cell and cell–matrix interactions
*RPL27*	Ribosomal protein L27	Catalyzes protein synthesis
*G3P/GAPDH*	Glyceraldehyde‐3‐phosphate dehydrogenase	Has both glyceraldehyde‐3‐phosphate dehydrogenase and nitrosylase activities
*FRIH/FTH1*	Ferritin heavy chain 1	Stores iron in a soluble, non‐toxic, readily available form
*PGS2/DCN*	Decorin	Involved in collagen fibril assembly
*TCTP/TPT1*	Translationally controlled 1	Regulator of cellular growth and proliferation
*NDUS5*	NADH: ubiquinone oxidoreductase subunit S5	Transfer of electrons from NADH to the respiratory chain
*UBIM/FAU*	FAU ubiquitin‐like and ribosomal protein S30 fusion	Fubi function unknown; ribosomal protein S30 displays antimicrobial activity

**TABLE 2:** Gene expression values of selected genes for validation as reference genes for the fat tissues of toothed whales

Seq_ID	Gene ID	Mean expression values (FPKM)	Standard deviations (± SDs)	Coefficient of variation (CV%)
MSTRG.20974	*ZC3H10*	3994.71	3257.74	81.55
MSTRG.35499	*RPL27*	696.19	783.52	112.54
MSTRG.4716	*LEG1/LGALS1*	1413.33	2483.88	175.75
MSTRG.24152	*FRIH/FTH1*	1643.36	758.31	46.14
MSTRG.11680	*G3P/GAPDH*	2456.70	3758.04	152.97
MSTRG.60098	*PGS2/DCN*	2892.99	4113.24	142.18
MSTRG.14006	*FRIL/FTL*	2317.56	1702.29	73.45
MSTRG.31040	*TCTP*	1518.02	712.44	46.93
MSTRG.33508	*NDUS5*	580.91	429.81	73.99
MSTRG.49478	*UBIM*	1385.68	1013.94	73.17

**FIGURE 3:**
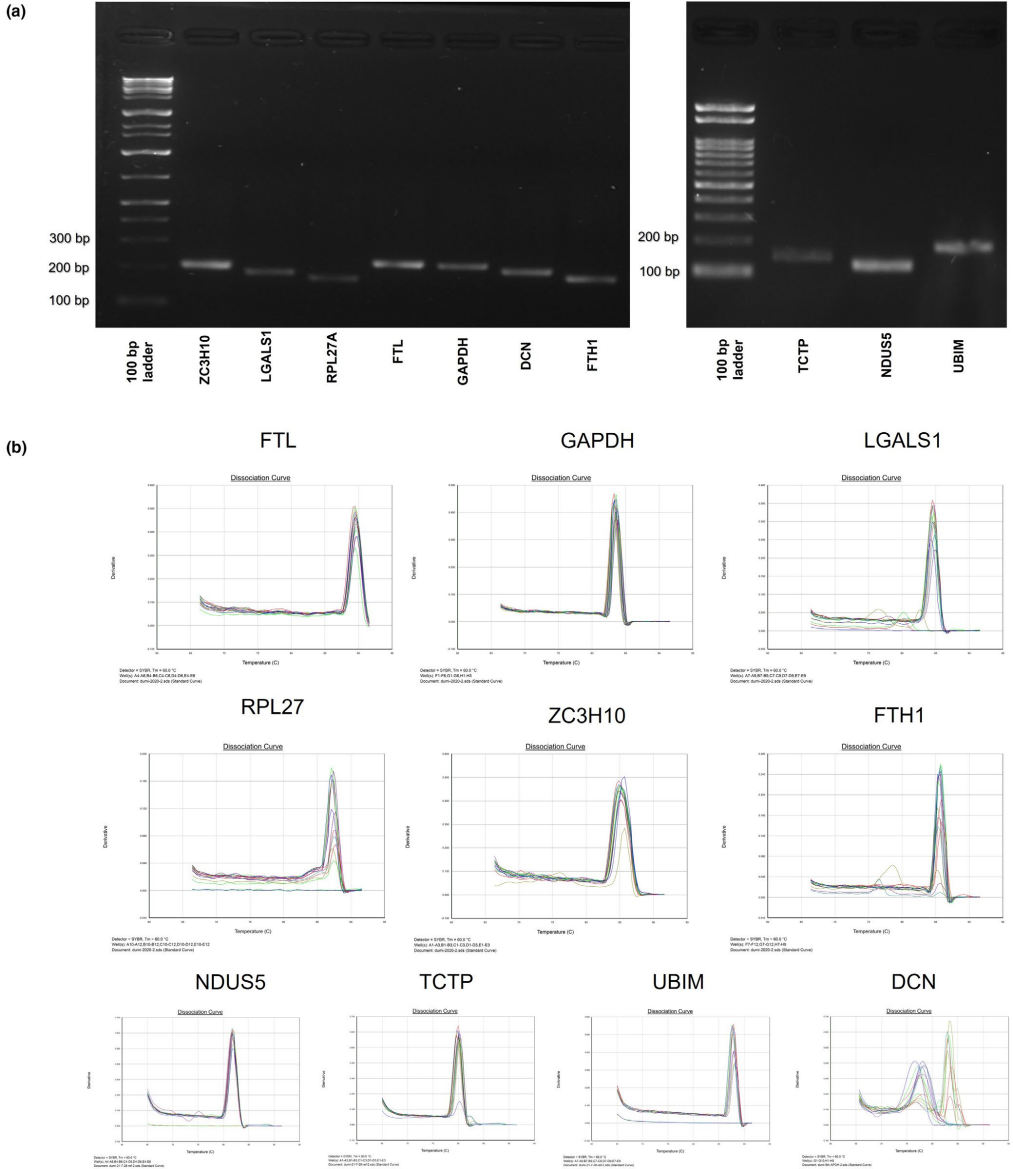
Results of PCR and qPCR analyses. (a) Image of 2% gel of agarose gel electrophoresis of selected 10 reference genes with 100 bp ladder. (b) RT‐qPCR dissociation curves for standards of all candidate reference genes tested in three 96‐well plates

The RT‐qPCR primers were designed using Primer3 (http://bioinfo.ut.ee/primer3‐0.4.0/) with consideration to primer length (~20 bp), product size (150–250 bp), and GC% content (40%–60%). Table 3 displays the primers. Before the RT‐qPCR analysis, the accuracy and specificity of all primers were checked by normal PCR conditions (using dNTP mix 1.6 µl, 10× Ex Taq buffer 2 µl, Ex Taq 0.1 µl, nuclease‐free water 13.3 µl, 0.5 µl from each forward and reverse primer, and 2 µl of cDNA as the template under the thermal cycle conditions of 95°C for 3 min, 35 cycles of 98°C for 10 s, 60°C for 30 s, 72°C for 30 s, and 72°C for 3 min final extension) and 2% agarose gel electrophoresis (Figure 3a).

**TABLE 3:** Primer information and amplification efficiencies for selected candidate genes

Gene ID	Primer sequence (5′–3′) forward/reverse	Amplicon size (bp)	Tm (°C)	Amplicon efficiency (%)	*R* ^2^	Slope
*ZC3H10*	GATGAGGCAGAGGTCCAAGT	234	58.4	82.259	.975	−3.836
	TGGCCATCGATGTTCCAGAT		56.3			
*FRIL/FTL*	GGCTTCTATTTCGACCGCGA	228	58.4	95.146	.995	−3.444
	GGCCTCGTTCAGGTTCTTCT		58.4			
*LEG1/LGALS1*	ATGGCTTGTTGTGACGCATT	182	54.3	94.357	.998	−3.465
	GGTCCTGGGCAAGTTTCTTG		58.4			
*RPL27A*	GGGTGAAAGGTAAGCGGAAG	167	58.4	122.321	.955	−2.882
	CTGAGGTGCCGTCATCAATG		58.4			
*G3P/GAPDH*	GTCGGAGTGAACGGATTTGG	218	58.4	94.882	.999	−3.451
	TGGAAGATGGTGATGGCCTT		56.3			
*FRIH/FTH1*	GCCTCCTACGTCTACCTGTC	197	60.4	75.421	.966	−4.097
	TTCTCCCAGTCATCACGGTC		58.4			
*PGS2/DCN*	AGAAGCTCTCCTACATCCGC	213	58.4	1936.576	.915	−0.764
	AATGAGGAGTGTTGGCCAGA		56.3			
*TCTP*	TCCTGGAGAAGCTAGACGCT	153	58.4	92.278	.972	−3.522
	CGAGCATGGTCTTTCCCCTT		58.4			
*NDUS5*	GGATAGAGTGCGCACATGGT	125	58.4	106.566	.939	−3.174
	CCTCTCGATGGCACTCAGAC		60.4			
*UBIM*	GCGGTATCCGCGTTTCAGTC	189	60.4	105.677	.954	−3.193
	CCAGGGTAGCCTCATCCTCTA		60			

### 2.4 Real‐time quantitative PCR (RT‐qPCR) and amplification efficiency

A RT‐qPCR was performed in 96‐well plates on an ABI 7300 Fast Real‐Time PCR system (ABI, Alameda, CA, USA) using the SYBR^®^ *Premix Ex* Taq™ kit (Cat. # RR820A, Takara Bio Inc., Japan). One qPCR reaction volume was 20 µl, and the conditions were as follows: 10 µl SYBR Premix Ex Taq (1×), 0.8 µl of 50 nM of both forward and reverse primers, 0.4 µl of ROX reference dye (50X), 6 µl of nuclease‐free water, and 2 µl of 100 ng of cDNA. All reactions and no template controls (NTCs) were conducted in triplicate. After 40 cycles, a melting curve analysis was conducted ranging from 55°C to 95°C, and cycle values (Ct data) were obtained from the relevant software of the RT‐qPCR system by automatically determining the threshold values. The dissociation curves of the RT‐qPCR indicate success of the qPCR experiment (Figure 3b).

**FIGURE 4:**
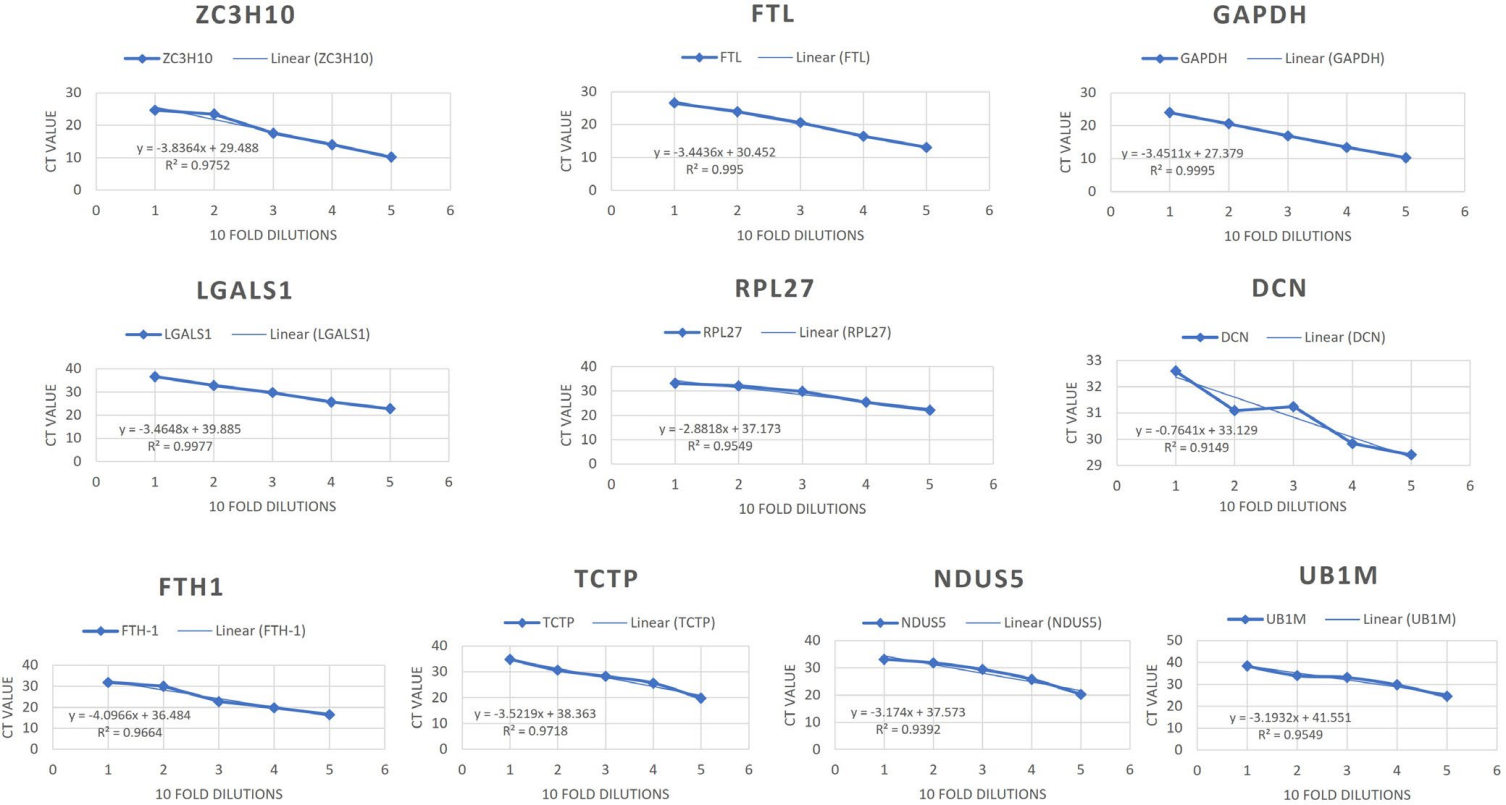
Efficiency of all selected genes based on standard curves (*Y* axis, mean Ct values of three replicates; *X* axis, 10‐fold changes from 1/10,000 [1] to 1 [5])

Standard curves were acquired for each primer pair by amplification in serial dilutions, such as 1:1, 1:10, 1:100, 1:1000, and 1:10,000, for all samples (Figure 4). The correlation coefficient (*R* ^2^) and the PCR amplification efficiency (*E*) for each gene was calculated from the slope of a standard curve, *E* = (10^(−1/slope)^ − 1) × 100% (Bustin et al., 2009). Based on the closer to 100% of *E* and closer to 1 of *R* ^2^ values, nine reference genes were selected for further analysis of gene stability to test for all biological samples in three replicates.

### 2.5 Gene stability analysis

A normality test for Ct data was conducted in the SPSS 17 statistical package (SPSS Inc, 2008) using the Kolmogorov–Smirnov test, and the normality of different genes was evaluated based on the *p*‐value. The statistical algorithms of Genorm (Vandesompele et al., 2002), NormFinder (Andersen et al., 2004), BestKeeper (Pfaffl et al., 2004), the comparative Delta CT method (Silver et al., 2006), and RefFinder (Xie et al., 2012) were used to determine the stability of candidate reference genes through an online version of RefFinder developed by Dr. Zhang's Lab (http://www.ciidirsinaloa.com.mx/RefFinder‐master/; Xie et al., 2012). The raw Ct data from each sample were used as an input file. Genorm performed a pair‐wise comparison among genes and calculated the expression stability value (*M*) for each gene. A low *M* value signifies higher stability. NormFinder also calculated an *M* value of gene stability based on the inter‐group and intra‐group variance and assigned the lowest *M* value to the most stable genes. The BestKeeper algorithm was applied to calculate the SD; genes were rated on the basis of variance, which also ascribes low SD and CV values to stable genes. The Delta CT values were obtained from pairwise differences between Ct values of reference genes to determine the stability of genes by repeatability among all samples. RefFinder considered the results of all of the above algorithms and generated a complete final ranking of the reference genes. The most stable reference genes were selected and subsequently analyzed for gene stability in 10 different tissues by using data from three replicates. Comprehensive graphs were prepared for the discussion.

## 3 RESULTS

In this study, a total of 30 samples (10 tissues from three individual Risso's dolphins) were employed to identify potential reference genes for the normalization of RT‐qPCR data across fat tissues and to validate transcriptomic data. Based on the RNA‐seq data (see Table A1), the study identified 10 highly expressed (FPKM > 1000) genes with CVs ranging from 46.14% to 175.75% (Table 2). Table 3 specifies the genes, primer information, amplicon length, and amplification information. Some genes, such as *FTL*, *ZC3H10*, *LGALS1*, *FTH1*, *DCN*, *TCTP*, *NDUS5*, and *UBIM*, have not been previously identified as reference genes. Gene sequence information of all candidate genes are provided in Table A2.

### 3.1 Primer specificity, amplification efficiency, and expression profiles

The specificity of each primer pair was confirmed by 2% agarose gel electrophoresis with a single band (Figure 3a). All genes presented a single melting peak in the qPCR, which indicates specific amplification (Figure 3b). The cDNA‐free NTC (negative control) samples did not display any melting curve product. These results suggest an absence of errors in the RT‐qPCR amplification. RT‐qPCR performance was good according to the coefficient (*R* ^2^) and efficiency (*E*) results (see Table 3 and Figure 4). As the standard curves illustrate, nine genes showed good slopes (nearly −3.4) and efficiencies (nearly 80%–120%), including six genes (90%–110% efficiency). Therefore, these genes were selected for gene stability analysis and to finally identify a potential reference gene for toothed whales. The *DCN* gene was not considered for further stability analysis because of its significantly low efficiency.

The expression profiles of the nine selected genes were tested by the mean quantification cycle (Ct) values for each sample with BestKeeper analysis (Table 4). The *FTH1* gene registered the lowest mean Ct value, while the highest expression and highest Ct value were recorded for *RPL27*. The expression of *TCTP* revealed wide variation among samples, whereas the expression of *LGALS1* was elevated and intense in different samples. However, to select an optimum candidate reference gene, additional analysis is recommended to further confirm the stability of genes based on Ct values.

**TABLE 4:** Distribution of mean Ct values of the nine selected candidate genes in all samples and standard deviations by BestKeeper

	Gene								
	*GAPDH*	*LGALS1*	*FTL*	*ZC3H10*	*RPL27*	*FTH1*	*TCTP*	*NDUS5*	*UBIM*
*n*	30	30	30	30	30	30	30	30	30
Geo mean (Ct)	15.38	22.28	13.96						
AR mean (Ct)	17	22.64	15.05	15.63	24.82	13.61	14.91	15.59	17.43
Min (Ct)	4.61	17.49	4.88	0	0	0	0	0	0
Max (Ct)	28.1	32.91	29.61	38.44	37.93	38.13	35.17	38.39	39.98
SD (± Ct)	5.42	3.28	4.38	6.25	11.58	8.44	12.03	11.74	13.18
CV (% Ct)	31.91	14.48	29.14	39.95	46.67	61.99	80.67	75.29	75.61
Min (*x*‐fold)	−1746.95	−27.74	−539.45	−1	−1	−1	−1	−1	−1
Max (*x*‐fold)	6740.54	1587.31	51550.24	3.73E+11	2.62E+11	3.02E+11	3.87E+10	3.59E+11	1.09E+12
SD (± *x*‐fold)	42.96	9.7	20.89	75.9	3066.25	346.71	4179.94	3414.87	9292.73

### 3.2 Validation of reference genes and identification of the most stable reference genes

**FIGURE 5:**
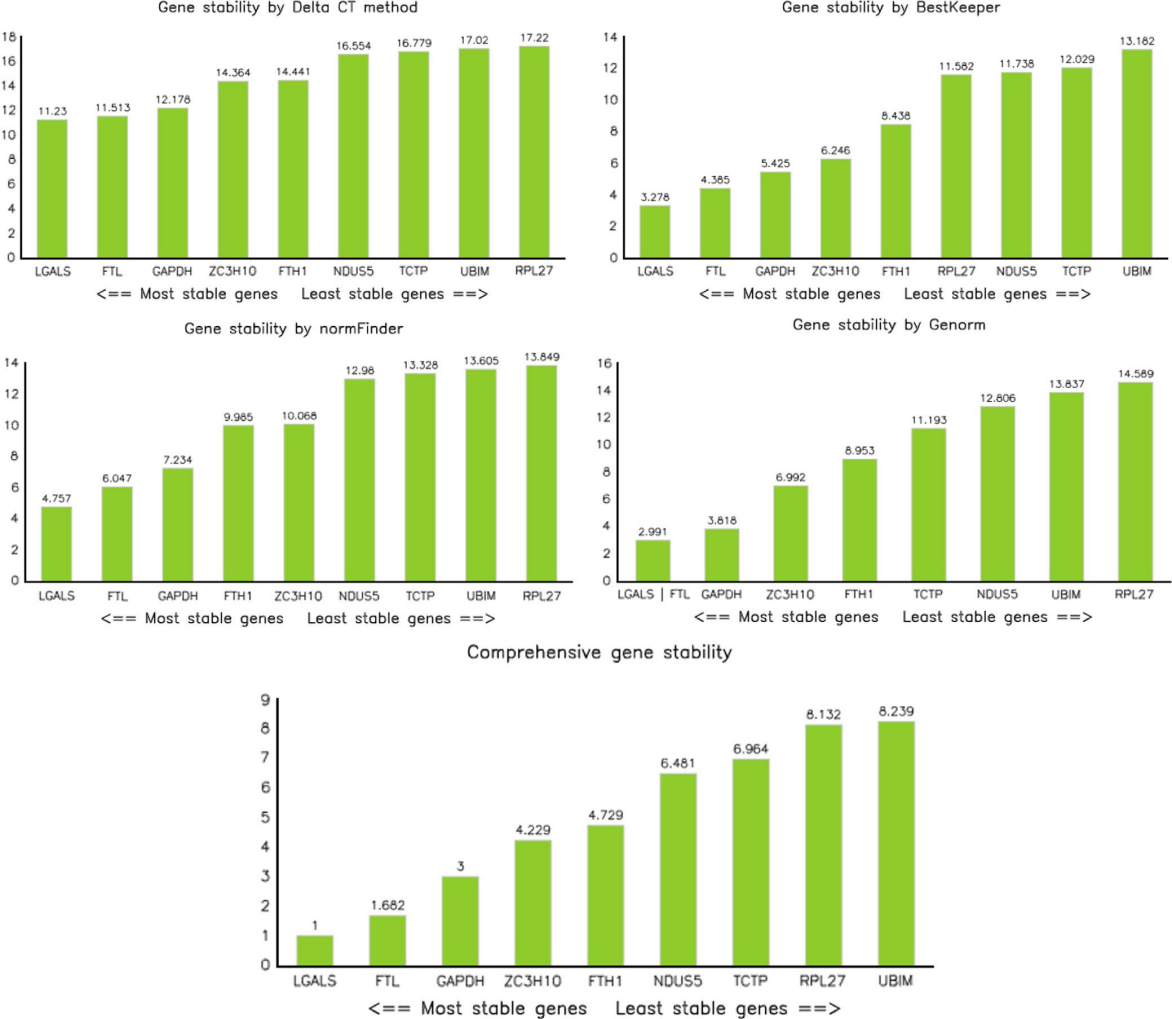
Histograms of gene stability analysis from four different algorithms (Delta CT, BestKeeper, NormFinder, and Genorm) and comprehensive gene stability

The Genorm statistical algorithm evaluated the stability of the selected reference genes by using the *M* value. The lowest value was assigned to the most stable gene, *LGALS1*, and *FTL*. The *RPL27A* gene had the highest *M* value, which suggests that it is the least stable (see Table 5 and Figure 5). NormFinder and Delta CT indicated the highest stability values for the *LGALS1* gene, *FTL*, and *GAPDH*, respectively. Likewise, BestKeeper yielded the highest stability values for the *LGALS1* gene, *FTL*, and *GAPDH*, respectively. The BestKeeper algorithm also calculated the Pearson correlation coefficient (*r*) among gene stability values, which differed significantly (*p* < .001). The lowest *r* value (.001) was obtained by *GAPDH*, *LGALS1*, and *FTH1*, while the highest value was registered by *UBIM* (.989). Finally, in the RefFinder comprehensive ranking, the *LGALS1* gene was identified as the most stable reference or housekeeping gene for transcriptome analysis, especially for different types of fat tissue in toothed whales (Table 6).

**TABLE 5:** Results of stability among three selected reference genes; four statistical algorithms (Delta CT, BestKeeper, NormFinder, and Genorm) were used for the estimation of stability values for 30 samples

Selected reference genes	RefFinder comprehensive ranking		Delta CT		BestKeeper		NormFinder		Genorm	
	Stability value	Rank	Average of SD	Rank	SD	Rank	Stability value	Rank	*M* value	Rank
*LGALS1*	1	1	11.23	1	3.28	1	4.76	1	2.99	1
*FTL*	1.68	2	11.51	2	4.38	2	6.05	2	2.99	1
*GAPDH*	3	3	12.18	3	5.42	3	7.23	3	3.82	3
*ZC3H10*	4.23	4	14.36	4	6.25	4	10.07	5	6.99	4
*FTH1*	4.73	5	14.44	5	8.44	5	9.99	4	8.95	5
*NDUS5*	6.48	6	16.55	6	11.74	7	12.98	6	12.81	7
*TCTP*	6.96	7	16.78	7	12.03	8	13.33	7	11.19	6
*RPL27A*	8.13	8	17.22	9	11.58	6	13.85	8	14.59	9
*UBIM*	8.24	9	17.02	8	13.18	9	13.61	9	13.84	8

**TABLE 6:** Summary of RefFinder gene ranking from all algorithms

Method	Ranking order (better–good–average)								
	1	2	3	4	5	6	7	8	9
Delta CT	*LGALS1*	*FTL*	*GAPDH*	*ZC3H10*	*FTH1*	*NDUS5*	*TCTP*	*UBIM*	*RPL27*
BestKeeper	*LGALS1*	*FTL*	*GAPDH*	*ZC3H10*	*FTH1*	*RPL27*	*NDUS5*	*TCTP*	*UBIM*
NormFinder	*LGALS1*	*FTL*	*GAPDH*	*FTH1*	*ZC3H10*	*NDUS5*	*TCTP*	*UBIM*	*RPL27*
Genorm	*LGALS1 | FTL*		*GAPDH*	*ZC3H10*	*FTH1*	*TCTP*	*NDUS5*	*UBIM*	*RPL27*
Recommended comprehensive ranking	***LGALS1***	***FTL***	***GAPDH***	***ZC3H10***	***FTH1***	***NDUS5***	***TCTP***	***RPL27***	***UBIM***

### 3.3 Stability of top three reference genes in different tissues

**FIGURE 6:**
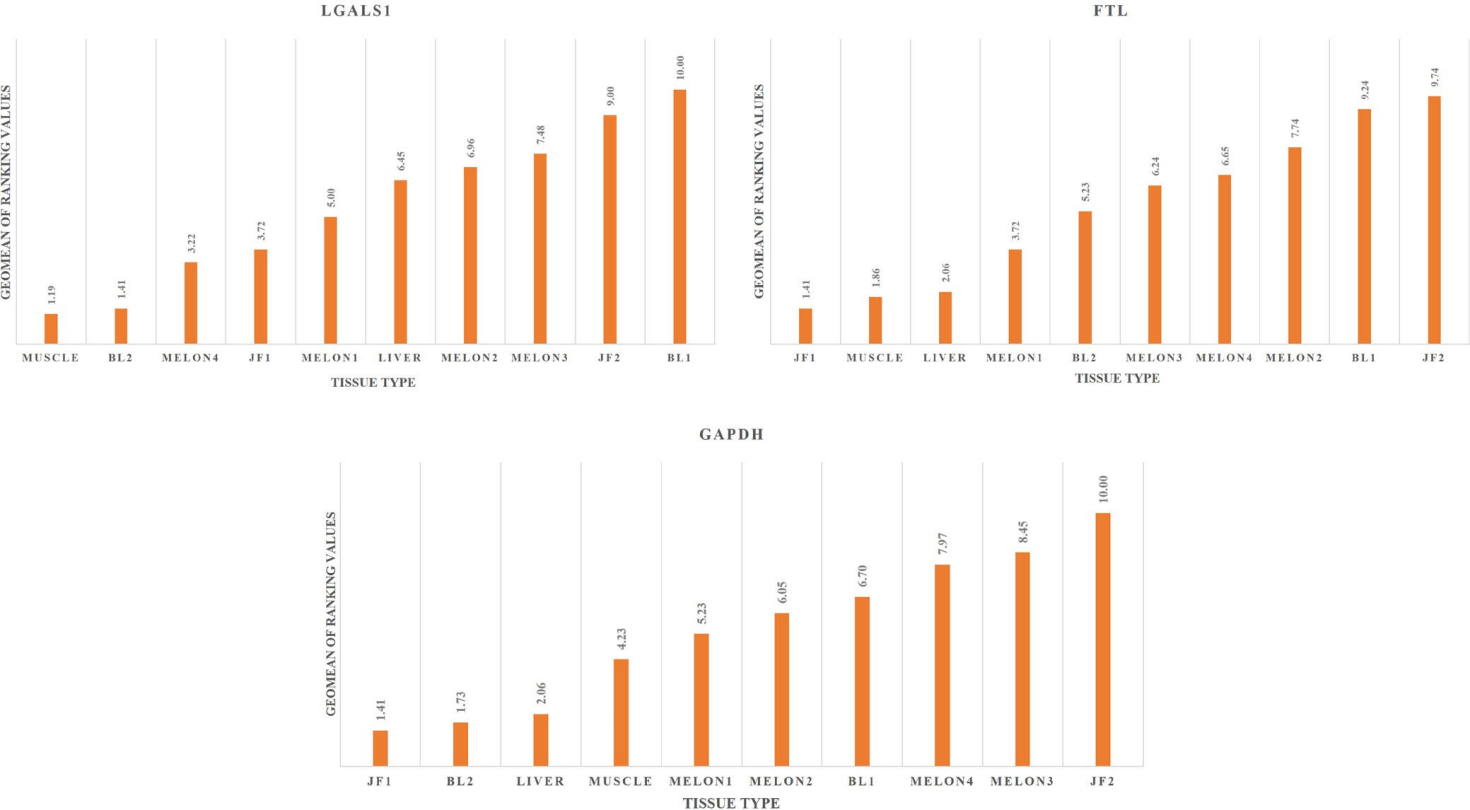
Comprehensive gene stability in 10 different tissues of Risso's dolphins (JF1, inner jaw fat; JF2, outer jaw fat; BL1, inner blubber fat; BL2, outer blubber fat). In this study, four parts of melon fat were identified as Melon 1–4

Based on the comprehensive gene stability values, the *FTL* gene was the most stable in the inner jaw fat sample (JF1), as it had the lowest stability value (1.414), and was the least stable in the outer jaw fat sample (JF2). Similar results were obtained for the *GAPDH* gene. However, interestingly, the *LGALS1* gene was more stable in muscle samples than in other fat tissues and was least stable in BL1 and JF2 (Figure 6).

## 4 DISCUSSION

In this research, all of the samples were collected at the same time. The analysis used 30 biological replicates of 10 types of tissue from three animals. All samples were preserved in RNAlater solution. Therefore, we believe that the total RNA of the various types of tissue sample was neither degraded nor significantly affected in a way that would change the Ct value of the RT‐qPCR technique. The selection of candidate reference genes for a study may depend on several factors, such as transcriptomic expression data, gene function, type of tissue, availability of samples, and quantity and quality of RNA extracted from the tissues. In this study, we specifically focused on finding a suitable reference gene in toothed whales using a variety of adipose tissues (melon, jaw fat, and blubber), muscle, and liver, which are mainly involved with aquatic adaptations. Liver and muscle tissues were used as controls, as they usually express many types of genes related to metabolism.

From comparative genomics analysis, it is evident that many genes are expressed in dolphins for aquatic adaptations, blubber, and fat storage (Mancia, 2018). The fat tissues of dolphins contain numerous fatty acids and fatty alcohol combinations; thus, the metabolism of these fats is still ambiguous. Considering all of these aspects, we determined candidate genes with the lowest CV percentage. Reference genes for qPCR can be identified through high‐throughput transcriptomic data by next‐generation sequencing (Bao et al., 2020; Gao et al., 2018; Pombo et al., 2018). All of our selected candidate reference genes exhibited a high level of expression by more than 500 FPKM values, and, according to multiple sources, their functions include regulatory and maintenance functions, metabolic processes, and catalysis in cells. Finally, this experiment was designed to detect 10 candidate reference genes, including eight uncommon reference genes and two well‐known HKGs. None of the tissues or genes is dependent on sex, which presumably precludes bias in the selected candidate genes due to the selection of only males for this study.

A range of ecological and evolutionary research has applied qPCR technologies during the last decade. One study has concluded that the qPCR approach is more sensitive and reproducible than the conventional polymerase chain reaction (cPCR) method for detecting target DNA in the prey–predation relationship (Yang et al., 2020). The qPCR assay has also been used in a study of the ecology and evolution of cryptic nematodes in a marine environment (Derycke et al., 2012). Furthermore, researchers have employed RT‐qPCR to study age‐ and sex‐related opsin gene expression in guppies compared with reference genes such as COI, β‐actin, and Myosin‐HC (Laver & Taylor, 2011). This study indicated that the variation in gene expression depends on various factors, including age and sex, as the adults of a guppy species expressed higher *LWS* compared with juveniles, which relates to reproductive fitness and male coloration. Therefore, the identification of a suitable reference gene for the normalization of qPCR‐based gene expression is important for ecological and evolutionary molecular studies of toothed whales. A stable and suitable reference gene is critical for correctly analyzing qPCR data (Linardic & Braybrook, 2021).

Cetaceans are successful marine mammals, and their aquatic adaptations are significant when compared with other mammals. In the transition from land to water, several genes were lost (e.g., *AANAT*, *ASMT*, and *MTNR1A*/*B*) in cetaceans (Espregueira Themudo et al., 2020; Huelsmann et al., 2019). The convergent and divergent evolution of mammalian genes has been discussed in a comparison of pigs and humans (Wei et al., 2018). Thus, RT‐qPCR based gene expression analysis is valuable for future research on the evolution of genes in aquatic mammals. Recent developments in next‐generation sequencing technologies and RT‐qPCR‐based approaches are currently being applied for diverse ecological examinations of non‐model animals (e.g., gene expression in eco‐immunology; Fassbinder‐Orth, 2014). The conditions of the aquatic environment, including temperature, salinity, pressure, and dissolved oxygen, can significantly affect toothed whales and their prey by changing their metabolism and lipid regulation. Assessing quantitative genetic parameters of wild marine vertebrates has also attracted immense interest (DiBattista et al., 2009). RT‐qPCR and transcriptomics are reliable approaches for the genetic analysis of quantitative or complex traits in organisms (e.g., genetic variance and heritability; Farries et al., 2019). Consequently, reference genes are also crucial for toothed whales’ quantitative and population genetics.

RT‐qPCR has become a widely used, accurate, and sensitive method to determine gene expression under various conditions and for many cell types in a range of organisms. Reference genes are essential for determining the level of expression of target genes, such as up‐regulation or down‐regulation by normalization (Freitas et al., 2019; Kozera & Rapacz, 2013). Several reference genes can be diagnosed by statistical algorithms, including NormFinder, BestKeeper, Genorm, and Delta CT (Xie et al., 2012). Therefore, the use of multiple HKGs in the normalization of genes of interest is currently a common practice. The *LGALS1* and *FTL* genes were identified as suitable novel genes for future functional genomics analysis of toothed whales from myriad ecological, evolutionary, and metabolic perspectives. The use of two reference genes for qPCR data analysis has been recommended in other research (Linardic & Braybrook, 2021). For the selection of stable genes, it is advised to employ more than one algorithm, which allows diverse analyses of data. Additionally, housekeeping genes also maintain normal cellular functions, which are confirmed in our experiment by the stable expression of all selected genes in muscle and liver tissues.

In our study, the *LGALS1* gene, one of the galectins, was ranked first by all algorithms in the stability tests. Functions of this gene include the modulation of immune responses (Nishi et al., 2008) and RAS protein signaling (Ruvolo et al., 2020). According to the recent updates, the *LGALS1* gene is highly expressed in fat tissues (NCBI, 2021); therefore, we believe it is a stable gene in the fats of toothed whales. Since, to our knowledge, no existing experiments have adopted the *LGALS1* and *FTL* genes as reference genes or HKGs, they are novel reference genes for marine mammals. The *FTL* gene is a protein, ferritin light chain that is mainly involved in the storage of iron in a soluble and nontoxic state in different cells (https://www.prospecbio.com/ferritin_human, accessed 2020/12/29). Likewise, the ERgene Python library for reference gene selection has highlighted the *FTL* gene as a stable endogenous reference gene for various studies (Zeng et al., 2020). Other research has experimentally identified TATA‐binding protein (*TBP*) and ATP Synthase Mitochondrial F1 Complex Assembly Factor 1 (*ATPF1*) as stable reference genes in the adipose tissue of mice (Almeida‐Oliveira et al., 2017). However, in our experimental samples, these genes were not highly expressed. The *GAPDH* gene was ranked third, although it is a widely used reference gene for many tissues of numerous animal species. The *RPS9* and *YWHAZ* genes have also been categorized as suitable reference genes from dolphin blood samples, although they did not include our samples from Risso's dolphins.

## 5 CONCLUSIONS

This study has contributed to research on toothed whales by systematically selecting and evaluating stable reference genes for RT‐qPCR using several types of tissue, including melon fat, jaw fat, blubber fat, liver, and muscle, from Risso's dolphins. Despite many limitations, the study has effectively checked 10 highly expressed candidate reference genes for efficiency. Two novel genes, *LGALS1* and *FTL*, have been identified as stable reference genes in toothed whales. The *LGALS1* gene was the most optimal reference gene and is therefore important for future ecological and evolutionary genomics studies on toothed whales. It also seems plausible that this study provides an accurate normalization factor for expression data on the genes of interest in various tissues of toothed whales.

## CONFLICT OF INTEREST

The authors declare that they have no conflicts of interest.

## AUTHOR CONTRIBUTIONS

**Jayan D. M. Senevirathna:** Conceptualization (equal); Data curation (equal); Formal analysis (equal); Investigation (equal); Methodology (equal); Validation (equal); Visualization (equal); Writing‐original draft (equal). **Ryo Yonezawa:** Data curation (equal); Formal analysis (equal); Methodology (equal); Validation (equal); Visualization (equal); Writing‐review & editing (equal). **Taiki Saka:** Data curation (equal); Formal analysis (equal); Methodology (equal); Validation (equal); Writing‐review & editing (equal). **Yoji Igarashi:** Conceptualization (equal); Data curation (equal); Formal analysis (equal); Investigation (equal); Methodology (equal); Validation (equal); Visualization (equal); Writing‐review & editing (equal). **Noriko Funasaka:** Conceptualization (equal); Data curation (equal); Investigation (equal); Methodology (equal); Resources (equal); Validation (equal); Writing‐review & editing (equal). **Kazutoshi Yoshitake:** Data curation (equal); Formal analysis (equal); Validation (equal); Visualization (equal); Writing‐review & editing (equal). **Shigeharu Kinoshita:** Investigation (equal); Validation (equal); Visualization (equal); Writing‐review & editing (equal). **Shuichi Asakawa:** Conceptualization (equal); Data curation (equal); Formal analysis (equal); Investigation (equal); Methodology (equal); Project administration (equal); Resources (equal); Supervision (equal); Validation (equal); Visualization (equal); Writing‐review & editing (equal).

## Figures and Tables

**TABLE A1:**
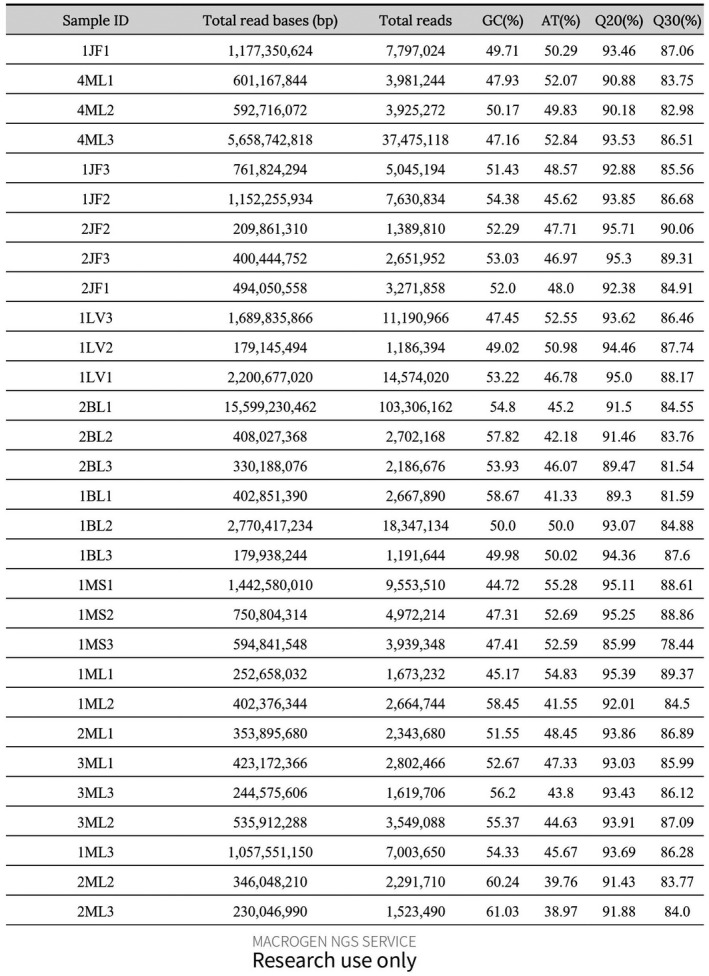
Statistics of RNA seq raw data after Illumina sequencing (1JF, inner jaw fat; 2JF, outer jaw fat; 1BL, outer blubber; 2BL, inner blubber; 1ML–4ML, four parts of the melon; MS, muscle; LV, liver)

## Data Availability

Selected transcriptomic data will be deposited in the DDBJ (DNA Data Bank of Japan) under the BioProject Accession PRJDB11720 and available on request from the corresponding author. All other data will also be publicly available with this article.

## APPENDIX 1

**TABLE A2:** Information on the transcriptomic sequence data of the selected candidate genes

Transcript ID	Gene ID	Prediction for the best matching	Transcript sequence
MSTRG.20974	*ZC3H10*	*Tursiops truncatus* zinc finger CCCH‐type containing 10 (*ZC3H10*), transcript variant X4, mRNA	TCTCGGCCTTAGCGCCATCTTCGGAGAAACCTCCGCGCCATGAGAGCGAAGTGGAGGAAGAAGCGAATGCGCAGGCTGAAGCGCAAAAGAAGAAAGATGAGGCAGAGGTCCAAGTAAACTTGTACACCCATGGAAGCCACAGGAGCAGAAACACGGGAAGCCAGAGGCCAGGGACGCTGGTACAAATTGTTGGACTGCTTGCCTACTGTCTAGAATTTGTCTCAGTGGATCTGGAACATCGATGGCCATTCTGATCGCCTCGACCGCCTTTGAGAGACCCACTTTGCTCGTATCAAAACGGCCCCTTTTGGCCCTTTGCCCTGGACCTTTTGACATACTGGACTAGTTCTATTCTCAGTTGTGGCTGAATGTAACATGTAACAATAAATCATATCTTTTGCTGTCTTAGCTGAAGAAAAAAAAAAAAAAAAAGT
MSTRG.14006	*FTL*	*Grampus griseus* FT mRNA for ferritin L subunit (*FTL*), complete cds	CCGCCATCTCTTCCCGCAGGGCTTCTATTTCGACCGCGACGATGTGGCTCTGGAGGGCGTGCGCCACTTTTTCCGCGAATTGGCCGAAGAGAAGCACGAGGGCGCCAAGCGTCTCTTGAAAATGCAAAACCAGCGCGGCGGCCGCGCCCTCTTCCAGGACGTGCAGAAGCCATCTCAAGATGAGTGGGGTAAAACTCAGGACGCTATGGAAGCCGCCATTAAAATGGAGAAGAACCTGAACGAGGCCCTTCTGGATCTGCATGCCCTGGCTTGTGCCCGCGCAGACCCCCACCTCTGCGACTTCCTGGAGAGCCACTTCCTAGATGAGGAGGTGAAACTCATCAAGAAGATGGGTGACCACCTGACCAACCTCCGCAGGCTGGCTGGTCCCCAGGCTGGGCTGGGCGAGTATCTCTTCGAAAGGCTCACCCTCAAGCACGATTAGGAGCCTCCGGAGCCCAGTGGCCTTTGAGGAGCCCCTCTGGTGTCAGGGCTTCTGCCTGAAGCCCCTCTCTGCAGCCAGTAGGCAGCTTGTTAACCACACTGGAGCCCTCTCCC
MSTRG.24152	*FTH1*	*Tursiops truncatus* ferritin heavy chain 1 (*FTH1*), mRNA	TAAGAGCCGGGCAAGAGTCACCTGCGCCACAGTCCTCGCGGAGAGTCGCCGCGGTTTCTTGCTTCAACAGTGCTTGGACGGAACCCGGCGCTCGTCCCCCGCTCCGGCCGGCCGCTCAGAGCCAGCCCTCGCCACCACCTCACGGCGCCCTCTGATCGCCCCAAGGTCCCCGCCGCCGTTACAGCGCCGCGCAGCCACCGCCACCGTTTCACCTCAGCCGCCCGCCATGACGACCGCGTCCCCCTCGCAGGTGCGCCAGAACTACCACCAGGACTCGGAGGCCGCCATCAACCGCCAGATCAACCTGGAGCTCTACGCCTCCTACGTCTACCTGTCCATGTCGTACTATTTTGACCGCGATGATGTGGCTTTGAAGAACTTTGCCAAATATTTTCTTCACCAATCTTCTGAGGAGAGGGAACATGCTGAGAAACTGATGAAGCTGCAGAACCAACGGGGTGGCCGAATCTTCCTTCAGGATATCAAGAAACCAGACCGTGATGACTGGGAGAATGGGCTGAATGCAATGGAATGTGCGCTACACTTGGAAAAAAGTGTGAATCAGTCACTACTGGAACTGCACAAACTGGCCACTGAGAAAAATGACCCCCATTTGTGTGACTTCCTTGAGACGCATTATCTGAATGAGCAGGTGAAATCCATCAAAGAATTGGGTGACCACGTGACCAACTTGCGCACGATGGGGGCCCCTGAATCTGGCATGGCACAGTATCTCTTTGACAAGCACACCCTGGGAAACAGTGATAATGAGAGCTAAGCCTTAGGCTGGCTTCCCGTAGCCACAGGGGTGACCTCCCTGGTCACCAAGGCAGTGCATGCATGTTTGGGTTACCTTACCTTTTCTGTAAGTTGTACCAAAATATCTACAAGTCCTTTCCTTAGTACTATTCCTTCAAATAAAGTAATTTGGTACCCA
MSTRG.4716	*LGALS1*	*Tursiops truncatus* galectin 1 (*LGALS1*), mRNA	GTCCAGTTAAAAGGGTGGGAGCGGGCTGTGGCCCATCTCTCGGGTAGTCTTCAGACAGCTGGTCCCGAAGTCCCAGGAACATCCTCCTCTCCTCAATCATGGCTTGTTGTGACGCATTTGGAGCCTGGTTCTCTCCATCTATTCAATGAGGCGATGGAGCACAGCACTCCACCACCCAGGCTACTCTGCATCTAAAATCCCCGGGCTAAACCGCAGAAACTTCTAGATCCTGTCTTCTAGAACCTCAGTCATGTTACAGACAAGAAACTTGCCCAGGACCACACAAAGAAGGAGAGTCTAGAACTTGCAGCCGGGTTCCTCAGCCTTCGGGTCGGCCTGGTCAGAGCAGGCCTGGTCTTCCCGGCGCGGGGCACGACCTCCCACCCTGCGTCCTCTAACCCGGCTGGGCGGGGTCTTGTCTGCGCAGGGTCTGGTCGCCAGCAACCTGAATCTCAAACCTGGGGAGTGCCTCAGAGTGCGGGGCGAGGTGCCCCCGGACGCCAAGAGCTTCGTGCTGAACATGGGCAAAGATGGCAACAACCTGTGCCTGCACTTCAACCCCCGCTTCGACGCGCACGGGGACACCAACACCATCGTGTGTAACAGCAAGGATGGCGGGGCCTGGGGGGCTGAGCAGCGGGAGTCCGTCTTCCCCTTCCAGCCTGGAAGTGTTGTGGAGGTGTGCATCTCCTTCGACCAGGCAGACCTAACCATCCAGCTGCCTGGTGGATACGATTTCAAGTTCCCCAACCGCCTCAACCTGGAGGCCATCAACTACCTGGCAGCCGATGGCGACTTCAAGATCAAGTGCGTGGCCTTTGAGTGAAGCCAGCCGGCCCATGGCCCCCAATAAAGGCAGCTGCCTCTGCTCCCCCTGAA
MSTRG.35499	*RPL27*	*Orcinus orca* ribosomal protein L27 (*RPL27*), mRNA	CCGAGGGGGCGGGGCAGGAAGTGAGGTCACCCTCCCCGGGGTGAAAGGTAAGCGGAAGTGCTCTTTCTTTCTCTGCTGTAGGCCCGAGAGGTTGCTGCCGACATGGGCAAGTTCATGAAACCCGGGAAGGTGGTGCTGGTCCTGGCCGGTCGCTACTCCGGACGCAAAGCGGTCATCGTAAAGAACATTGATGACGGCACCTCAGACCGACCCTACAGCCATGCTCTGGTGGCTGGAATTGATCGCTATCCCCGCAAAGTGACAGCTGCCATGGGCAAGAAGAAAATTGCCAAGAGGTCAAAGATCAAGTCTTTCGTGAAAGTTTATAATTATAATCATCTCATGCCTACAAGGTACTCTGTGGATATCCCCTTGGACAAAACTGTTGTCAACAAGGATGTCTTCAGAGACCCTGCTCTCAAANNNNNN
MSTRG.11680	*GAPDH*	*Tursiops truncatus* glyceraldehyde−3‐phosphate dehydrogenase (*GAPDH*), mRNA	ATAAATTCCGCCTGCAGCCTTCCCCTGCGCTCTCTGCTCCTACCCGTTCGACAGGCAGCCGTGTGTTGTGTGCAGTGCCAGCTGCGTCCCGGAGACAAGATGGTGAAGGTCGGAGTGAACGGATTTGGCCGTATTGGGCGCCTGGTCACCAGGGCTGCTTTTAACTCTGGCAAAGTGGACATTGTCGCCATCAATGACCCCTTCATTGACCTTCACTACATGGTCTACATGTTCCAGTATGATTCCACCCACGGCAAGTTCCATGGCACAGTCAAGGCTGAGAACGGGAAGCTTGTCGTCAATGGAAAGGCCATCACCATCTTCCAGGAGCGAGATCCCGCCAACATCAAATGGGGTGATGCTGGCGCTGAGTACGTGGTGGAGTCCACCGGCGTCTTCACCACCATGGAGAAGGCCGGGGCTCACTTGAAGGGTGGAGCCAAGAGGGTCATCATCTCTGCCCCTTCCGCCGATGCCCCCATGTTCGTCATGGGCGTGAACCATGAGAAGTATGACAACCACCTCAAGATCGTCAGCAATGCCTCCTGCACCACCAACTGCTTGGCCCCCCTGGCCAAGGTCATCCATGACCACTTCGGCATCGTGGAGGGACTCATGACGACGGTCCATGCCATCACTGCCACCCAGAAGACCGTGGATGGCCCCTCTGGGAAGCTGTGGCGTGATGGCCGAGGCGCTGCCCAGAACATCATCCCTGCTTCCACTGGTGCTGCCAAGGCCGTGGGCAAGGTCATCCCTGAGCTGAACGGGAAGCTCACTGGCATGGCTTTCCGCGTCCCCACCCCCAACGTGTCCGTCGTGGATCTGACCTGCCGCCTGGAGAAACCTGCCAAATACGAGGACATCAAGAAGGTGGTGAAGCAAGCGTCCGAAGGGCCCCTCAAGGGCATCCTGGGCTACACTGAGGACCAGGTTGTCTCCTGTGACTTCAACAGTGACACCCACTCTTCTACCTTCGATGCTGGGGCTGGCATCGCCCTCAACGACCACTTTGTCAAGCTCATTTCCTGGTACGACAATGAATTTGGCTACAGCAACAGGGTGGTGGACCTCATGGTCCACATGGCCTCCAAGGAGTAAGAGTCCCTGGACCACCAGCCCCAGCAGGAACACAAGAGGAAGAGAGAGGTCCTCCGCTGCTGGGGAGTCCTTGCCCCAACTCCGTCCTCCAACACACTTGAGAATCTCCCGACCTCCATGCGGTTTCCATCCCAGACCCCCTGAGGAAGGGGAGGAGGAGGGGCTTAGGGAGCCCTACCTTGTCATGTACCATCAATAAAGTACACTGCACCCAG
MSTRG.31040	*TCTP*	*Tursiops truncatus* tumor protein, translationally controlled 1 (TPT1/*TCTP*), mRNA	CGCAGCGGGAGATGACGTAGGGGGACGTGCCCTCTATATGAGGTTGGGGAGCGGCAGAGTCGGCCTTTTCCGCCCGCTCCCCCCTCCCCCCGAGCGCCGCTCCGGCTGCACTGCGCTCGCTCTGAGCTCCGTGCTCCTAAGCTAGTGCCACCGTTGTCTCCCTCCAGTCGCCATCATGATCATCTACCGGGACCTCATCAGCCATGATGAGATGTTCTCCGACATTTACAAGATCCGGGAGGTTGCGGACGGGCTGTGTCTGGAGGTGGAGGGGAAGATGGTCAGTAGGACAGAGGGTAACATTGATGACTCGCTCATTGGTGGAAATGCCTCCGCTGAAGGCCCCGAGGGCGAAGGTACTGAAAGCACGGTAATCACTGGTGTGGATATTGTCATGAACCATCACTTGCAGGAAACCAGCTTCACAAAAGAAGCCTACAAGAAGTACATCAAAGATTACATGAAGTCAATCAAAGGGAAGCTTGAAGAACAGAGACCAGAAAGAGTAAAACCTTTTATGACAGGGGCTGCAGAACAAATCAAGCATATCCTTGCTAATTTCAAAAACTATCAGTTCTTTATTGGTGAAAACATGAATCCAGATGGCATGGTTGCTCTGCTGGACTACCGTGAGGATGGTGTGACCCCATATATGATTTTCTTTAAGGATGGTCTAGAAATGGAAAAATGTTAACGAATTCGGCTGTTTGGATCTATCACCTGCTCGTCATAACCGGCTGGCTGCTGCTTTTCATCCACACAACACCGGGACTTAGACAAATGGGACTGATGTCATCTTGAGCTCTTCGTTTTGATGTTGATTTGTTTGGAGCGGAGGCATTGTTTTTGAGGAAAAAAAACCATGTCATCCTTCCTGGAGAAGCTAGACGCTGGTTGTAGACCACTACTAGAAAGCATAAGACCATCTTCAGATAACTTCTGCAATGAAAACCACTTCCGGGACTTGGAATATAAACAAGAATCAACAGTAATTGAGTTGAAATAAAGGGGAAAGACCATGCTCGTAGCAGTGCCAGCATTTGAAGTGGATGTCTTACACATTTCATCACCTACAAATGGAAGTAGTTAACTCTGGAAGAAATTACCAAAAGAATAAAAAGAGACTTAACACAGTTGGAAGCAAAGTATTGTCTCAGCTTATTTACAAAGCAAGTAGAGCCAAGTTCTAGAGAAATCTAGACGTCTCCTGGAATACTTGTGTCAAACTTCTTGGAACAGGGTTGGCAAACTGGCTTGTCCTACCACCTGTTTTTGTGTGACTAAGGTGGTGCTTACATTTTTAAGTGGTTCAAAATAGCTTACGGGATGTGAAAAAATACAAAATCGAAGTTTGTG
MSTRG.33508	*NDUS5*	*Lagenorhynchus obliquidens* NADH:ubiquinone oxidoreductase subunit S5 (*NDUFS5*/*NDUS5*), mRNA	GCGCTGCAAGCGTCGCGCTGCGGGCTTCCTTTGCGGCAGGCCTCATTGTCACCTGAGGATCTTCTAGTAGCGGAGTCAGCAAGGAGTCAAGGACACAGCTGCTGGACGCCATGCCTTTCTTTGATGTGCAGAAAAGGCTGGGTCTTGACTTAGACCGCTGGATGACAATCCAGAGTGCTGAGCAGCCTCTCAAGATTCCAGGTCGATGCCATGCTTTTGAAAAAGAATGGATAGAGTGCGCACATGGTATTGGTGGTATTCGCGCGCAGAAAGAGTGCAAGATAGAATATGATGATTTCGTAGAGTGTCTACTTCGACAGAAAACGATGAAACGTCTGAGTGCCATCGAGAGGCAGCGGGATAAGCTAATAAAGGAAGGGAAGTACACACCTCCACCTCAGCACCTGGGCAAGGAGGATCCTTGACCCTGAGCAGAGCAGCTGCCGGTGGCTGGAGGCTGATTTTCACGTTCTCTCTTCTCTGCTGGAAACTTTACGTACTGAAAACCCTTTTGTGAAAGTGTCTAAAAATAAAGGATTGCTCTCTCCTATTGGTTCTATA
MSTRG.49478	*UBIM*	*Tursiops truncatus* FAU, ubiquitin like and ribosomal protein S30 fusion (*FAU*/*UBIM*), mRNA	GGCCTTGTCGGTCGCTCAGCAGTGACGTGACACGCAGCAGGCGACGAGGCCGCACCCGCCCTAGCTCCTTCCTCTTTCTCGACTCCATCTTCGCGGTAGCGGCAGCGGTATCCGCGTTTCAGTCGCCAACATGCAGCTCTTTGTCCGCGCCCAGGAGCTACACACTCTCGAGGTGACCGTCCAGGAGACGGTCGCCCAGATCAAGGCTCATGTAGCCTCGTTGGAGGGCATCGCTCCAGAAGATCAAGTCCTGCTTATGGCAGGCACACCCCTAGAGGATGAGGCTACCCTGGGCCAGTGTGGGGTGGAGGCTCTGAGCACTCTGGAAGTAGCCGGCCGCATGCTTGGAGGTAAAGTCCATGGTTCCCTGGCCCGAGCCGGGAAAGTAAGAGGTCAGACACCGAAGGTGGCCAAACAAGAGAAAAAGAAGAAGAAGACGGGCCGGGCCAAGAGGCGTATGCAGTACAACCGGCGCTTTGTCAACGTTGTGCCTACCTTTGGCAAGAAGAAGGGCCCCAATGCCAACTCTTAAGTGTGTTGTTATCTTGGCTTTCTCTAATAAAGTCACTTACCCAATTA
MSTRG.60098	*DCN*	*Tursiops truncatus decorin (DCN), transcript variant X1, mRNA*	AGTCAAGTTGCTCCACTGCTATACTACAGAAGAAGTTTAGAATCTTAAGCagatgaaaaaggaataaagcatttaaatggggagaaaaaaaggccGATAAAATTTCTGGCTACAATACAAGAGACATAtcattaccatatgatctaatgTGGGTGTCAGCCGGATTGTGTTCATTGAGGGAAACCTTATTTTTTAACTGTGCTATGGAGTAGAAGCAGGAGGTTTTCAACCTAGTGACAGTCACAGAGCAgcacctgccccctcctcctttccacaCCTGCAAACTCTTTTGCCTGGGCTGAATATTTAGTGTAATTACATCTCAGCTTTGAGGGCTCCTGTGGCAGATCCCCGGATTAAAAGGTTCCTTGGTTGTGAAAATACATGAGATAAATCATGAAGGCAACTTTCATCTTCCTCTTGCTCGCACAAGTTTCCTGGGCTGGACCGTTTCAGCAGAAAGGCTTATTTGACTTTATGCTGGAAGATGAGGCTTCTGGGATAGGCCCGGACGACCGCTTTCACGAAGTTCCTGAATTAGAGCCTCAGGTGCCAGTGTGCCCCTTCCGCTGTCAATGCCATCTTCGAGTTGTCCAGTGTTCTGATTTGGGTCTGGAAAAAGTGCCAAAAGATCTTCCCCCTGACACTGCACTGCTGGACCTGCAAAACAACAAGATAACTGAAATCAAAGATGGAGACTTTAAGAACCTGAAGAACCTTCACACGTTGATTCTTATCAACAACAAAATTAGCAAAATCAGTCCTGGGGCATTTGCACCTTTGGTGAAATTGGAACGACTTTATCTGTCCAAGAATCAACTGAAGGAATTGCCAGAGAAAATGCCCAAAACTCTTCAGGAGCTGCGTGTCCATGAGAACGAGATCACCAAAGTGCGAAAGTCTGTGCTCAATGGATTGAACCAGTTGATTGTCGTAGAACTTGGCACCAACCCTCTGAAGAGCTCAGGCATTGAAAATGGAGCCTTCCAGGGAATGAAGAAGCTCTCCTACATCCGCATTGCTGACACCAATATAACCACCATCCCTCAAGgtcttcctccttcccttactGAATTACATCTTGATGGCAACAAAATCACCAAAGTTGATGCAGCTAGCCTGAAAGGACTGAATAATTTGGCTAAGTTGGGACTGGGTTTCAACAGCATCTCTGCTGTCGACAATGACTCTCTGGCCAACACTCCTCATTTGAGGGAACTTCATTTGAACAACAACAAGCTTATCAAAGTGCCTGGTGGGCTGGCGGATCATAAGTACATCCAGGTTGTCTACCTTCATAACAACAACATCTCTGCAATCGGCCCTAACGACTTCTGCCCACCTGGATACAACACCAAAAAGGCTTCTTATTCCGGGGTGAGCCTTTTCAGCAACCCAGTCCAGTACTGGGAGATCCAGCCATCCACCTTCCGATGTGTCTACGTGCGCTCTGCCATTCAGCTCGGAAACTACAAGTAGTTCCGAAGAAAACCCTCGTTTTTATAACCTGGCAAAATCCCGTTAACGtcactgctcaaaaaaaaaaaaaaaaaaaaaaaaaaaaaaaaaaaatgaaagctagaTACTGGAAACCTAACTGCAATGTGGATGTTTTCCCCACATGACTTATTATGCGTAAAGCCAAATATGCAGTTTAAATAATTgcctacaataaaaaaaaaatattttgcctgCCATTTTCAGAATCCTGTTTTGAAGTTTATTATTGATGTTAACTGAGCTATTGCAGATATTCTTATTTCACTAAATGTAAAATTTGGAGTAAACAATTTAGTAGAGCTTTTAATTTCCcgaagaaatcaataaaaatggtATCAATCTTCTGTAACTCATAGGGGGGGTTAGCGCCTTTGACATAGTCTAGAAATAATGTATTCATCCCCATTATTACTGATTAAGCCTCATTTGAGTGTGTAAATTCAATACAGGCTATGTAAAATTTTTGCtaatgtcattattttgaaaaaaaataaattcacaagtacattttaaattactattgTATAAAAGCGTAATTGTAAATCTTCCCTAACCTCAATTTTCTGAAGAGAGTAAACATTGCTTTCTTAATAAAAACAGCACACCTTT
